# Supplementary material for: Digital electron diffraction – seeing the whole picture
Source: Acta Crystallogr A. 2013 May 21;69(Pt 4):427–34. doi: 10.1107/S0108767313010143 (PMC3686228; doi:10.1107/S0108767313010143)
Supplement: Supplementary file 1 [file a-69-00427-sup1.pdf]

Supplementary information to 'Digital' Electron Diffraction - Seeing the Whole Picture

RichardBeanland, [a*]Paul JThomas[b] David I Woodward,[a] Pamela A Thomas[a] and Rudolf A Roemer[a]

[a]Department of Physics, University of Warwick, Coventry CV4 7AL, UK, and [b]Gatan UK Ltd, 25 Nuffield Way, Abingdon, Oxon, OX14 1RL, UK. E-mail: r.beanland@warwick.ac.uk

The electron beam used in this work was typically 15nm full-width half maximum (FWHM), as shown below.

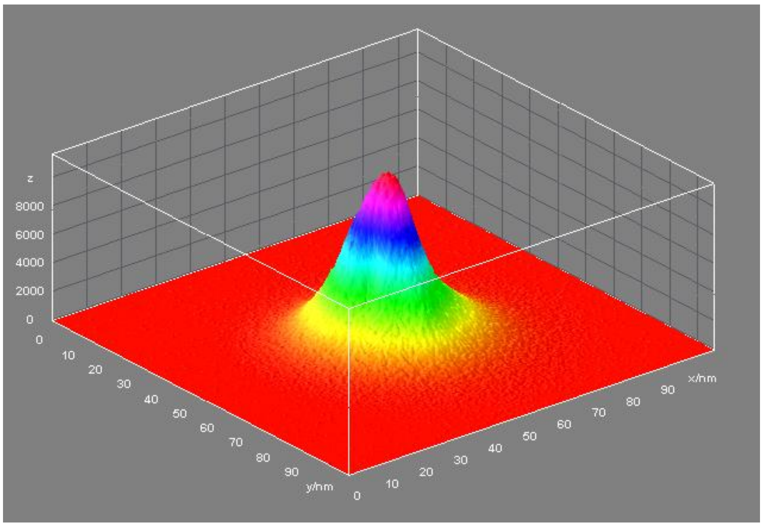

Large beam tilts can result in significant displacement of the beam, mainly due to spherical aberration of the pre-field objective lens. Although this can be to some extent compensated using feedback in the microscope, this fails quite quickly and it is best to use a look-up table for beam position in combination with computer control of beam shift. (Koch, 2011). Video 'Uncorrected.avi' shows the variation in beam position while the beam is tilted with no compensation. Video 'Corrected.avi' shows the beam position with compensation. Some small changes in beam shape are visible at large beam tilts. The width of the image is 250nm.

References

Koch, C. T. (2011). *Ultramicroscopy* **111**, 828-840.
